# CYP2D6 Genotype Predicts Tamoxifen Discontinuation and Prognosis in Patients With Breast Cancer

**DOI:** 10.1200/JCO.19.01535

**Published:** 2019-12-04

**Authors:** Wei He, Felix Grassmann, Mikael Eriksson, Erik Eliasson, Sara Margolin, Linda Thorén, Per Hall, Kamila Czene

**Affiliations:** ^1^ Department of Medical Epidemiology and Biostatistics, Karolinska Institutet, Stockholm, Sweden; ^2^ Department of Laboratory Medicine, Clinical Pharmacology, Karolinska Institutet and Karolinska University Hospital, Stockholm, Sweden; ^3^ Department of Oncology, South General Hospital, Stockholm, Sweden; ^4^ Department of Clinical Science and Education Södersjukhuset, Karolinska Institutet, Stockholm, Sweden

## Abstract

**PURPOSE:**

To examine the association between CYP2D6 genotype, discontinuation of tamoxifen therapy, and prognosis for breast cancer.

**PATIENTS AND METHODS:**

We conducted a prospective-retrospective study linking data from a clinical breast cancer register, the Swedish Prescribed Drug Register, and self-reported questionnaires. We genotyped CYP2D6 in 1,309 patients with breast cancer who were treated with tamoxifen and were diagnosed from 2005 to 2012; they were categorized as poor, intermediate, normal, or ultrarapid CYP2D6 metabolizers. We investigated whether metabolizer status was associated with tamoxifen discontinuation and prognosis for breast cancer using Cox regression analysis.

**RESULTS:**

The 6-month discontinuation rates of tamoxifen among poor, intermediate, normal, and ultrarapid CYP2D6 metabolizers were 7.1%, 7.6%, 6.7%, and 18.8%, respectively. A U-shaped association was found between CYP2D6 metabolizer status and breast cancer–specific mortality, with adjusted hazard ratios of 2.59 (95% CI, 1.01 to 6.67) for poor, 1.48 (95% CI, 0.72 to 3.05) for intermediate, 1 (reference) for normal, and 4.52 (95% CI, 1.42 to 14.37) for ultrarapid CYP2D6 metabolizers.

**CONCLUSION:**

Both poor and ultrarapid CYP2D6 metabolizers of tamoxifen have a worse prognosis for breast cancer compared with normal metabolizers after receiving a standard dose of tamoxifen. This U-shaped association might call for individualized tamoxifen dosage.

## INTRODUCTION

Adjuvant tamoxifen therapy reduces breast cancer mortality by 31%.^1^ However, tamoxifen effectiveness vary widely between individuals.^2^ Tamoxifen is a drug that requires metabolic activation by hepatic cytochrome P450 2D6 (CYP2D6) to elicit full pharmacologic activity because the corresponding metabolites 4-OH-tamoxifen and endoxifen exhibit much higher binding affinity to the estrogen receptor than the parent compound.^2^^-^^4^ Early evidence has shown that poor CYP2D6 metabolism could predict worse clinical outcomes among patients with breast cancer who are treated with tamoxifen.^4^ Several studies have confirmed^5^^-^^7^ and some have contradicted^8^^,^^9^ this observation. As a result, conflicting recommendations have been made regarding the use of CYP2D6 genotype for individualizing dosages of tamoxifen.^10^^,^^11^

Compared with the controversial but relatively extensive studies on poor CYP2D6 metabolism, few studies have been able to investigate the association between ultrarapid CYP2D6 metabolism and tamoxifen response. Several lines of evidence have suggested that ultrarapid CYP2D6 metabolizers have a higher endoxifen level than normal metabolizers,^11^^-^^13^ a finding that has raised the hypothesis that ultrarapid CYP2D6 metabolizers may have better (at least no worse) tamoxifen response after receiving a standard dose of tamoxifen.^11^ On the basis of this hypothesis, the 2018 Clinical Pharmacogenetics Implementation Consortium (CPIC) guideline for CYP2D6 and tamoxifen therapies recommends that patients with ultrarapid CYP2D6 metabolism use a standard dose of tamoxifen (strength of recommendation: strong).^11^ This recommendation, however, has not considered the possibility that women with ultrarapid tamoxifen metabolism may have more severe tamoxifen-related adverse effects^14^ and thus be more likely to discontinue tamoxifen.^11^^-^^13^ Our aim is to test the hypothesis that both poor and ultrarapid CYP2D6 metabolizers of tamoxifen have a worse prognosis compared with women who are normal metabolizers.

## PATIENTS AND METHODS

### Study Population

Our study included data from two Swedish Breast Cancer Cohorts namely, Linné-bröst 1 (LIBRO1) and Karolinska mammography project for risk prediction of breast cancer (KARMA). LIBRO1 is a case-only cohort composed of 5,175 women diagnosed with breast cancer between January 1, 2001, and December 31, 2008, in Stockholm, Sweden.^15^^,^^16^ KARMA is a cohort study initiated in 2011 and is composed of 70,877 women who attended mammography screening or received a clinical mammography at 4 hospitals in Sweden.^17^ Questionnaires, study materials, and follow-up strategy were similar for both studies because LIBRO1 was the pilot study for the KARMA study. The similarities enabled us to combine data from LIBRO1 and KARMA as we had done before.^15^^,^^18^^,^^19^

**FIG 1.**
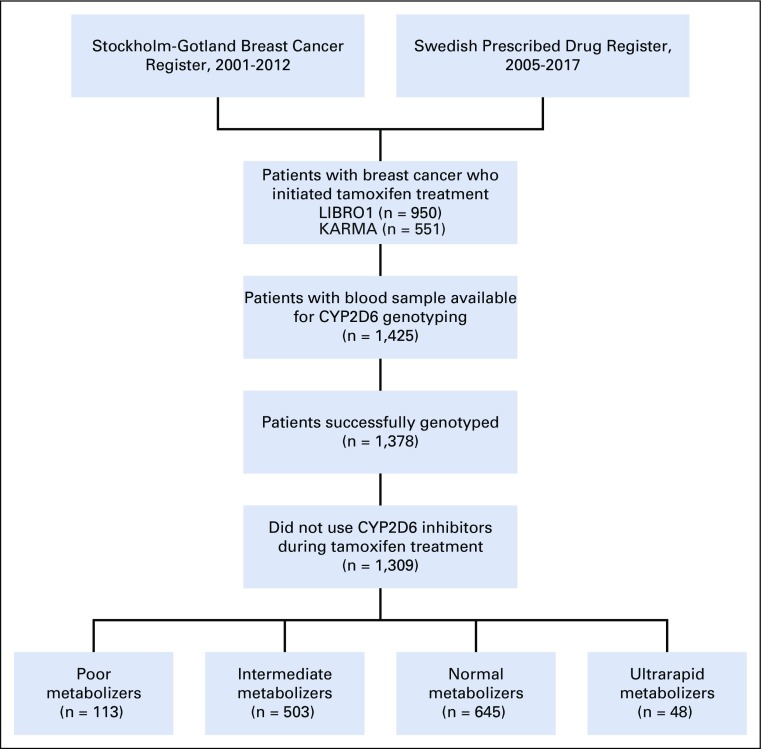
Flowchart of study participants.

We included all patients diagnosed with stages I to III breast cancer between 2005 and 2012 who had ever used tamoxifen (Fig 1). Only women diagnosed after 2005 were chosen because the Swedish Prescribed Drug Register was established in 2005 and register-based information on tamoxifen use was not available before that year. By linking to the Swedish Prescribed Drug Register, we identified 1,501 patients with breast cancer who initiated tamoxifen treatment without switching to aromatase inhibitors. Among them, we excluded 76 patients with inadequate or no DNA samples, 47 patients for whom CYP2D6 genotyping failed, and 69 patients who had ever filled one or more prescriptions of moderate-to-potent CYP2D6 inhibitors^20^ (paroxetine, fluoxetine, bupropion, duloxetine, thioridazine, perphenazine, pimozide, quinidine, ticlopidine, terbinafine, or cinacalcet) during their tamoxifen therapy, leaving a total of 1,309 women for the final analyses (Fig 1). Both LIBRO1 and KARMA were approved by the Regional Ethical Review Board in Stockholm, Sweden. All women provided written informed consent.

### CYP2D6 Genotype and Phenotype

DNA was extracted from peripheral blood, and we genotyped 14 single nucleotide variants representing the most common CYP2D6 alleles (*2, *2A, *3, *4, *6, *7, *8, *9, *10, *14, *17, *29, *35, *41A) to maximize phenotype prediction by using polymerase chain reaction–based TaqMan allelic discrimination assays on a custom TaqMan OpenArray Genotyping Plate (CYP2D6 alleles, variants, and assays are listed in Appendix Table A1, online only). CYP2D6 gene deletions (*5), duplications, or multiplications were determined by using TaqMan gene copy number assay according to the manufacturer’s instructions (Applied Biosystems, Foster City, CA). When no allele-defining sequence variation was identified, CYP2D6*1 was assigned as the wild-type (reference) allele. All variants were in Hardy-Weinberg equilibrium (except CYP2D6*4, *6, *10) and matched the frequencies expected in a white population (Appendix Table A1).^21^

CYP2D6 activity score was determined for each patient according to the methods introduced by Gaedigk et al.^22^ In short, a value was assigned to each allele, and the activity score was the sum of the values assigned to each allele.^11^ CYP2D6 activity score was then used to classify patients with breast cancer as poor (PM, activity score = 0), intermediate (IM, activity score = 0.5 or 1.0), normal (NM, activity score = 1.5 or 2.0), or ultrarapid (UM, activity score > 2.0) CYP2D6 metabolizers.^11^

### Definitions of Covariates

Variables that may have potentially affected tamoxifen discontinuation and mortality were selected as covariates. Information on age, menopausal status, tumor size, lymph node involvement, progesterone receptor status, human epidermal growth factor receptor 2 (HER2) status, tumor grade, radiotherapy, and chemotherapy was retrieved from the Regional Quality Register for Breast Cancer (2005-2006) and the National Quality Register for Breast Cancer (2007-2012). Information on comorbidities used to calculate the Charlson comorbidity index was retrieved from the Swedish Patient Register. Information on education, body mass index, tobacco use, and parity was retrieved from questionnaire surveys. Detailed information about the questionnaire survey can be found in our previous publications.^16^^,^^23^^,^^24^

### Outcome Definitions

#### Use of symptom-relieving drugs.

Use of symptom-relieving drugs was defined as filling at least one prescription of the corresponding drugs within 90 days of tamoxifen initiation, including antinauseants (Anatomical Therapeutic Chemical [ATC] code A04), anxiolytics (ATC code N05B), analgesics (ATC code N02), and hot flash medications (ATC codes N06AB, N06AX16, N03AX12, C02AC01, N02CX02, and S01EA04).^25^^,^^26^ A window of 90 days after tamoxifen initiation was selected because tamoxifen-related adverse effects, if present, usually occur during this time period.^27^ Women who used the corresponding symptom-relieving drugs within 90 days before tamoxifen initiation were excluded from the analyses.

#### Tamoxifen discontinuation.

Tamoxifen discontinuation was defined as having any interval between 2 consecutive tamoxifen dispenses exceeding 180 days during the follow-up.^23^ In Sweden, a 3-month supply is the maximum that is allowed to be dispensed. Therefore, an interval of more than 180 days indicates that at least 2 dispenses have been missed, thus resulting in a shortage of tamoxifen for the patient. More information regarding tamoxifen discontinuation can be found in our previous publications.^18^^,^^23^^,^^24^^,^^28^

Patients were observed from the time they received their first prescription for tamoxifen, and they were censored at death, emigration, contralateral breast cancer, local recurrence, distant metastasis, end of study period (December 2018), or completion of 5-year follow-up, whichever came first.^23^ Date of tamoxifen discontinuation was defined as the date on which the last prescription of tamoxifen was filled plus the days of supply corresponding to the last prescription. Time to tamoxifen discontinuation was defined as the time interval between the date of tamoxifen initiation and the date of tamoxifen discontinuation.

#### All-cause and breast cancer–specific mortality.

Patients were observed from their first prescription for tamoxifen until death, emigration, or end of study period (December 2018), whichever came first, to define all-cause and breast cancer–specific mortality. Time to death was defined as the time interval between the date of tamoxifen initiation and the date of death. Information on cause of death was retrieved from the Swedish Cause of Death Register.

### Statistical Analyses

χ^2^ tests were used to assess whether baseline characteristics and the use of symptom-relieving drugs differed by CYP2D6 metabolizer status. Kaplan-Meier analyses were used to investigate the association of CYP2D6 metabolism with treatment discontinuation. The Schoenfeld residual test was used to check the proportionality assumption of the Cox regression analysis. A violation of the assumption was indicated for the association between ultrarapid CYP2D6 metabolism and tamoxifen discontinuation. The time-dependent hazard ratios (HRs) for ultrarapid CYP2D6 metabolism were therefore estimated by using flexible parametric survival models.^29^

We used the delayed-entry Cox regression analysis to investigate the association of CYP2D6 metabolism with all-cause and breast cancer–specific mortality. The delayed-entry model was used to adjust for the fact that women had their blood drawn at a median time of 2.1 years (25% to 75% interquartile; 1.34 to 2.98 years) after their first date of tamoxifen use.

Sensitivity analyses were conducted to assess the robustness of our findings. We stratified our analyses by menopausal status, we conducted additional analyses to investigate tamoxifen discontinuation rates by use of symptom-relieving drugs, and we conducted additional analyses to investigate breast cancer mortality rates by tamoxifen discontinuation. We used SAS, version 9.4 (SAS Institute, Cary, NC) and STATA, version 15.1 (STATA, College Station, TX), for all of the analyses. All statistical tests were two-sided, and statistical significance was defined as *P* < .05.

## RESULTS

### Patient Characteristics

Table 1 shows the baseline characteristics by CYP2D6 metabolizer status. The prevalence of poor and ultrarapid CYP2D6 metabolizers was 8.6% and 3.7%, respectively. There were no significant differences in baseline and tumor characteristics between CYP2D6 metabolizer status groups. The median age at cancer diagnosis was 58.1 years (range, 25.2 to 88.7 years), and the median follow-up period was 10.4 years (range, 1.1 to 13.4 years).

**TABLE 1.**
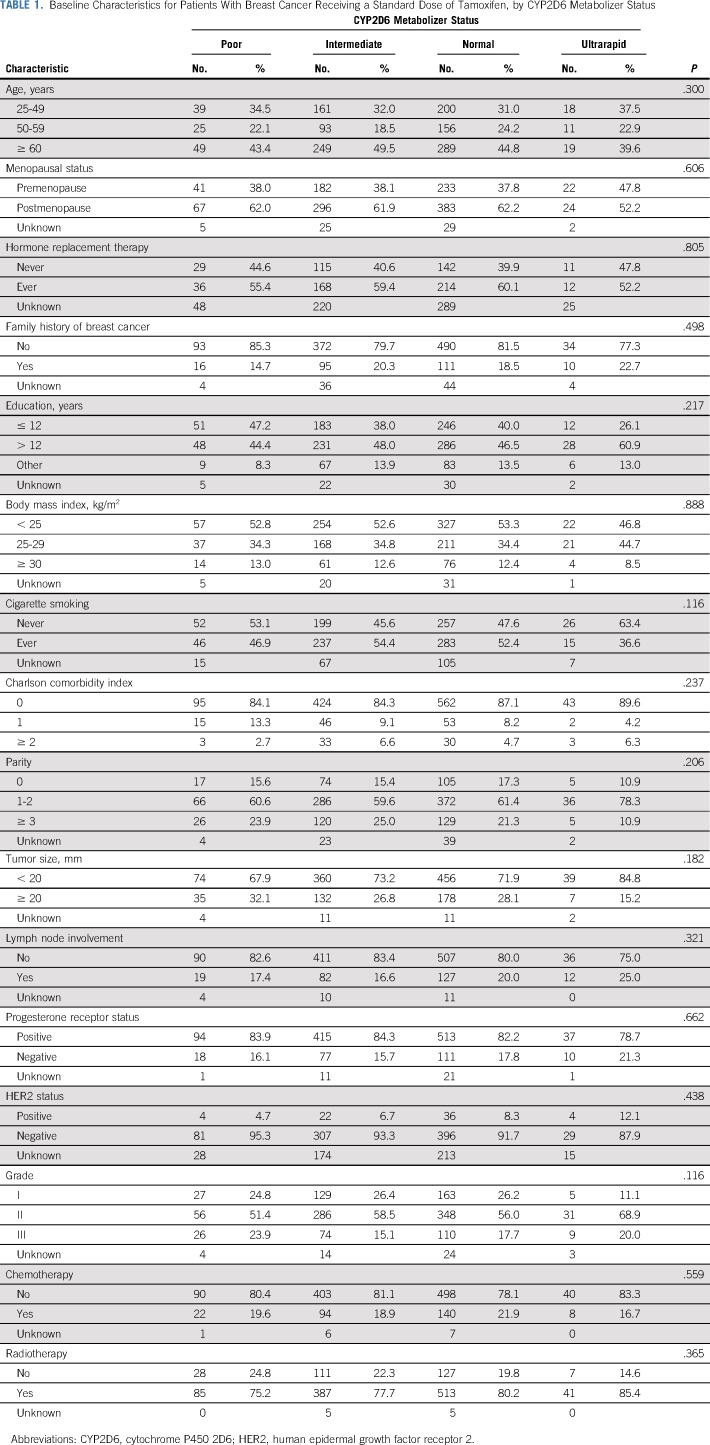
Baseline Characteristics for Patients With Breast Cancer Receiving a Standard Dose of Tamoxifen, by CYP2D6 Metabolizer Status

### Tamoxifen Metabolism by CYP2D6 Metabolizer Status and Use of Symptom-Relieving Drugs

**FIG 2.**
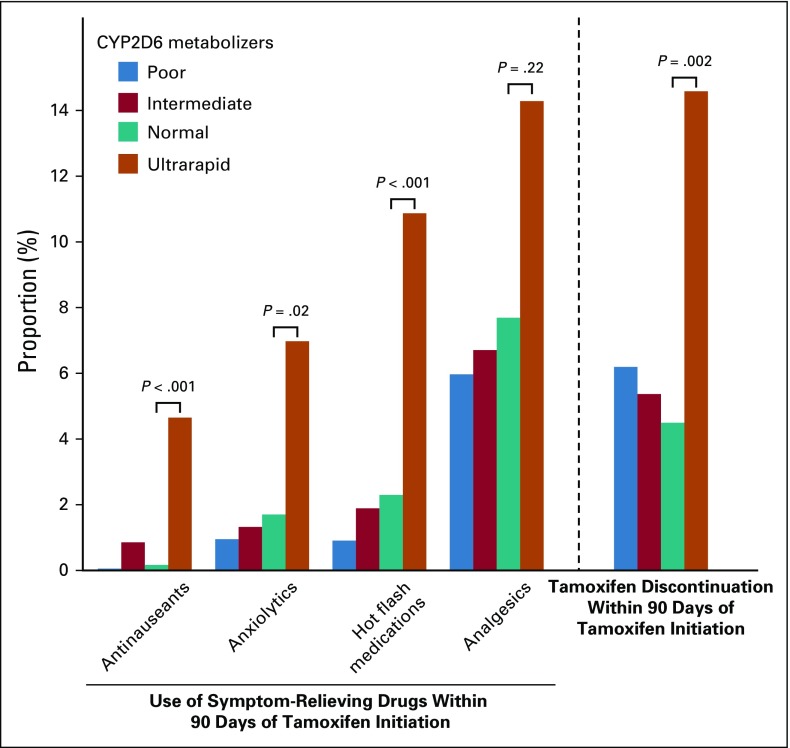
Use of symptom-relieving drugs and tamoxifen discontinuation among patients with breast cancer receiving standard dose of tamoxifen, by CYP2D6 metabolizer status. The use of symptom-relieving drugs was defined as any prescription of corresponding drugs within 90 days of tamoxifen initiation. Women with any prescription of corresponding symptom-relieving drugs 90 days before tamoxifen initiation were excluded from the analyses. No significant difference was found for poor or intermediate metabolizers compared with normal metabolizers for the use of symptom-relieving drugs or tamoxifen discontinuation.

Figure 2 shows the association between CYP2D6 metabolizer status and the use of symptom-relieving drugs within 3 months after receiving the standard dose of tamoxifen. Ultrarapid CYP2D6 metabolizers were significantly more likely than normal metabolizers to use symptom-relieving drugs, including antinauseants, anxiolytics, and medications for relief from hot flashes. No significant difference was found for poor or intermediate metabolizers compared with normal metabolizers for the use of symptom-relieving drugs.

### Tamoxifen Metabolism by CYP2D6 Metabolizer Status and Tamoxifen Discontinuation

**FIG 3.**
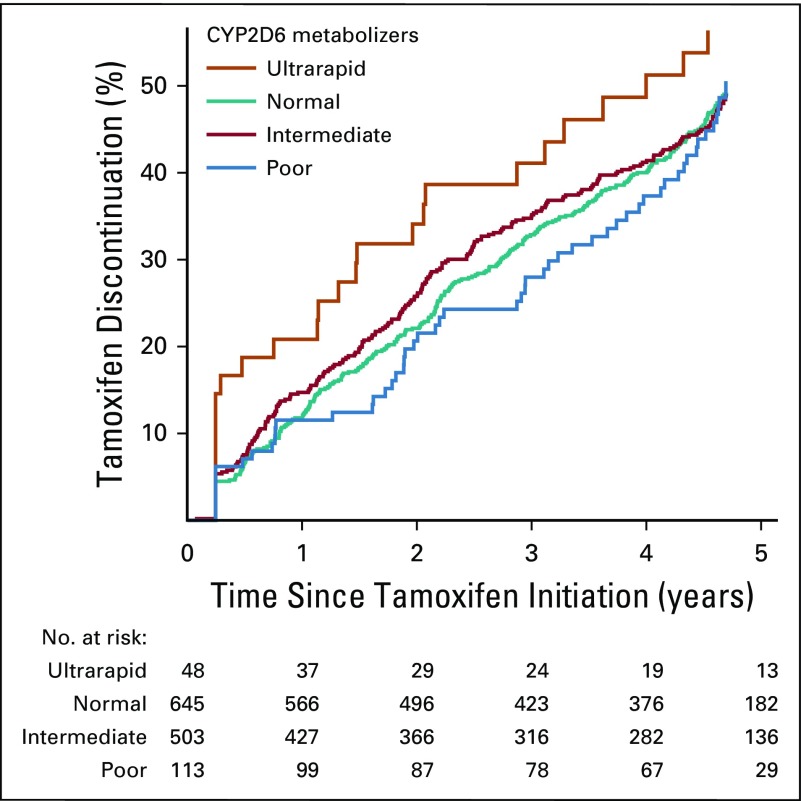
Treatment discontinuation among patients with breast cancer receiving standard dose of tamoxifen, by CYP2D6 metabolizer status.

Figure 3 shows the association between CYP2D6 metabolizer status and tamoxifen discontinuation. The 6-month tamoxifen discontinuation rates among ultrarapid CYP2D6 metabolizers was 18.8% (95% CI, 10.2% to 32.9%), which was significantly higher than the 6.7% (95% CI, 5.0% to 8.9%) observed among normal metabolizers. Flexible parametric survival analysis (with adjusting for all the variables listed in Table 1) shows that, compared with normal metabolizers, ultrarapid CYP2D6 metabolizers were more likely to discontinue their treatment within the first 6 months after tamoxifen initiation (HR at month 6, 2.06; 95% CI, 1.11 to 3.82), but not afterward. No significant difference was found for poor or intermediate metabolizers compared with normal metabolizers for tamoxifen discontinuation.

### Tamoxifen Metabolism by CYP2D6 Metabolizer Status and Mortality

Table 2 shows the association between CYP2D6 metabolizer status and mortality. Ultrarapid CYP2D6 metabolizers had a significantly higher breast cancer–specific mortality (HR, 4.52; 95% CI, 1.42 to 14.37) compared with normal metabolizers after adjusting for all variables listed in Table 1. When comparing poor CYP2D6 metabolizers to normal metabolizers, a more than twofold increased breast cancer–specific mortality rate was seen (HR, 2.59; 95% CI, 1.01 to 6.67) after adjusting for all the variables listed in Table 1.

**TABLE 2.**
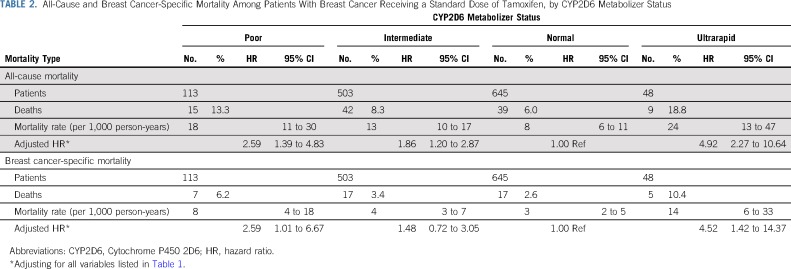
All-Cause and Breast Cancer-Specific Mortality Among Patients With Breast Cancer Receiving a Standard Dose of Tamoxifen, by CYP2D6 Metabolizer Status

### Sensitivity Analyses

**FIG A1.**
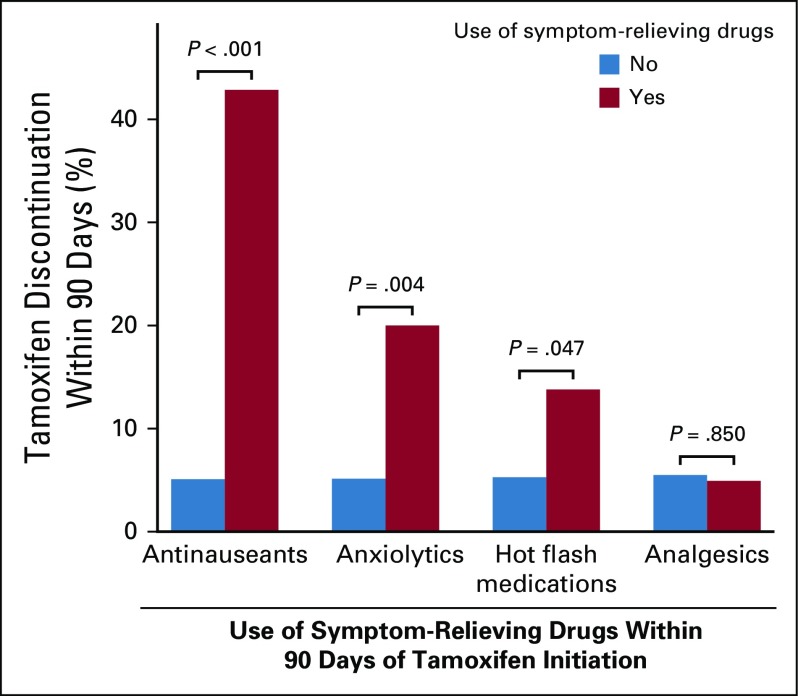
Tamoxifen discontinuation among patients with breast cancer receiving a standard dose of tamoxifen, by use of symptom-relieving drugs. The use of symptom-relieving drugs was defined as any prescription of corresponding drugs within 90 days of tamoxifen initiation. Women with any prescription of corresponding symptom-relieving drugs 90 days before tamoxifen initiation were excluded from the analyses.

**FIG A2.**
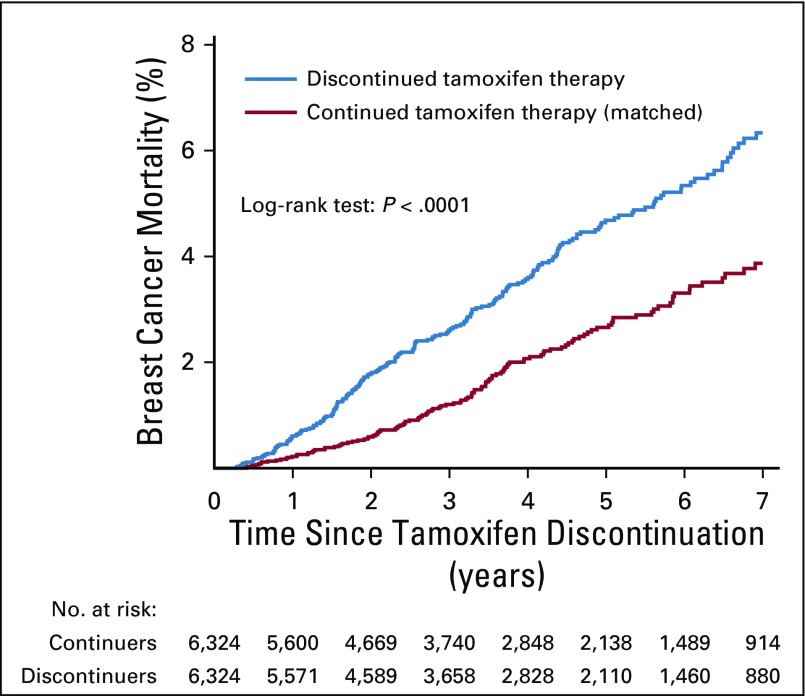
Breast cancer mortality among patients receiving standard dose of tamoxifen designated as those who discontinued tamoxifen therapy versus those who continued tamoxifen therapy (matched). Data from the Swedish Cancer Register, Swedish Prescribed Drug Register, and Swedish Cause of Death Register were linked, and patients were observed until December 2015. The source population is all women diagnosed with stages I to III breast cancer in Sweden from 2005 to 2015. Those who continued therapy were 1:1 matched to those who discontinued therapy on age, calendar year of cancer diagnosis, tumor size, and lymph node involvement. By definition, no death occurred within 180 days after tamoxifen discontinuation.

Stratified analyses by menopausal status provided results similar to those in the main analysis (Appendix Table A2, online only). Additional analyses revealed higher tamoxifen discontinuation rates among users of symptom-relieving drugs (except for painkillers; Appendix Fig A1, online only) and higher breast cancer mortality among tamoxifen discontinuers (compared with matched continuers; Appendix Fig A2, online only).

## DISCUSSION

Both poor and ultrarapid CYP2D6 metabolizers of tamoxifen have a worse prognosis for breast cancer compared with normal metabolizers. In contrast to normal metabolizers, ultrarapid metabolizers were significantly more likely to use symptom-relieving drugs and discontinue tamoxifen use.

Ultrarapid CYP2D6 metabolizers can have an endoxifen level of around 80 nmol/L at standard doses of tamoxifen,^30^ which is higher than the accepted therapeutic level of 35 nmol/L or more.^12^ Whether a higher endoxifen level translates into a better prognosis is debatable.^31^ Previous studies have hypothesized that the concentration of endoxifen should be within a therapeutic range to balance tamoxifen efficacy and adverse drug reactions.^32^^-^^35^ Exceeding this range may have negative therapeutic effects, leading to life-threatening or intolerable adverse effects.^4^^,^^32^^-^^35^

Our study showed that ultrarapid CYP2D6 metabolizers are more likely than normal metabolizers to discontinue tamoxifen use in an early phase of therapy. The result is novel but not surprising because previous studies have shown that treatment-related adverse effects are more likely to cause early rather than late tamoxifen discontinuation.^36^

Patients with breast cancer who are ultrarapid CYP2D6 metabolizers are usually recommended to use a standard dose of tamoxifen.^11^ However, ultrarapid CYP2D6 metabolizers cannot benefit from tamoxifen if they do not take it. The high 6-month discontinuation rate of 18.8% could translate to one in six ultrarapid CYP2D6 metabolizers discontinuing treatment within the first 6 months after tamoxifen initiation. This suggests that the current CPIC guideline, without considering treatment discontinuation of ultrarapid CYP2D6 metabolizers, may be misleading.

The quality of evidence for the association between poor CYP2D6 metabolism and mortality—which is deemed necessary to practice truly evidence-based medicine—received a grade of weak in the 2018 CPIC guideline.^11^ This weak evidence may be partially a result of the fact that most previous studies on poor CYP2D6 metabolizers have defined their comparison (reference) group by combining ultrarapid metabolizers and normal metabolizers into one group.^8^^,^^9^^,^^11^ Such a combination dilutes the association between poor CYP2D6 metabolism and tamoxifen outcomes. In this study, classifying ultrarapid metabolizers as normal metabolizers reduced the HR for breast cancer mortality among poor metabolizers from 2.59 to 2.15. This decreased HR consequently increased the power needed to detect a statistically significant association, a problem further exacerbated by the fact that many CYP2D6 studies have been underpowered because of a limited number of patients and events.^11^ Failure to discriminate ultrarapid from normal CYP2D6 metabolizers, which is common because copy number variations were not commonly determined in previous CYP2D6 studies,^8^^,^^9^^,^^11^ might thus partially explain the previous inability to detect an association between CYP2D6 and tamoxifen response.^11^

Failure to adjust for tamoxifen adherence has been hypothesized as another factor that may explain the mixed results on poor CYP2D6 metabolism.^20^^,^^37^ It has been suggested that poor CYP2D6 metabolizers may have better tamoxifen adherence.^38^ However, we did not see a difference in adherence or use of symptom-relieving drugs among poor, intermediate, and normal metabolizers.

The third potential explanation for the mixed results on poor CYP2D6 metabolism is the use of DNA from different sources in different studies.^39^ Two trials have previously reported a null association between poor CYP2D6 metabolism and breast cancer outcomes.^8^^,^^9^ However, the validity of the two studies has been questioned because those trials used tumor DNA.^39^

It has not previously been shown, with sufficient power and long-term follow-up, that poor and ultrarapid CYP2D6 metabolizers are more likely than normal metabolizers to die as a result of breast cancer. This is an important message because millions of women globally are prescribed tamoxifen for the treatment or prevention of breast cancer.^20^^,^^40^ The proportion of poor and ultrarapid CYP2D6 metabolism has been estimated to be 5.4% and 3.1% in Europe,^35^ 1.9% and 4.6% in the Americas, and 0.4% and 21.2% in Oceania, respectively.^41^ Therefore, the effect of poor and ultrarapid CYP2D6 metabolism on tamoxifen treatment is unlikely to be negligible; it might be associated with a less optimal therapy in hundreds of thousands of patients with breast cancer.

Our results suggest that the higher mortality observed among ultrarapid CYP2D6 metabolizers may at least in part be a result of tamoxifen discontinuation. However, because of the low proportion of ultrarapid CYP2D6 metabolizers in the population (3.7%), our study is underpowered to determine the underlying causes for the higher mortality observed among ultrarapid CYP2D6 metabolizers. Thus, studies with larger sample size and measurements of serum tamoxifen metabolites are needed.

Our study has several limitations. First, potential misclassification of ultrarapid metabolizers is possible because among patients with more than two CYP2D6 gene copies, the duplicated alleles were assigned on the basis of the most common allele in two-copy carries. However, such misclassification will diminish rather than strengthen the observed association. Our sensitivity analyses, which were restricted to patients with the same activity score for both alleles (ie, those with homozygous genotype across the gene and thus without potential misclassification because of genotype uncertainty) showed consistent results (HR, 6.16; 95% CI, 1.85 to 20.53 for breast cancer mortality). Second, in our study, survival bias was possible because blood was drawn at a median of 2.1 years (25% to 75% interquartile, 1.34 to 2.98 years) after tamoxifen initiation. However, we believe this bias is very small because 99% of patients with stage I to III breast cancer who received tamoxifen in Stockholm survived the first 2 years after tamoxifen initiation during our study period.

There is currently no agreement on the test for tamoxifen metabolizer status in the clinical setting. Since 2006, oncologists at Mayo Clinic have offered CYP2D6 testing to patients who have been prescribed tamoxifen,^42^ although others are hesitating.^42^ Our results may help accelerate the transition from evidence to guidelines and enable clinicians to make informed recommendations on CYP2D6 genotyping. However, because this is the first study with sufficient power and detailed drug prescription data to demonstrate an effect of ultrarapid CYP2D6 metabolism on tamoxifen outcomes, confirmation of our findings in another cohort is warranted.

In conclusion, our study demonstrates that both poor and ultrarapid CYP2D6 metabolizers of tamoxifen have a worse prognosis compared with normal metabolizers. Our findings thus strengthen the need to revisit the current guideline that recommends patients with breast cancer use a standard dose of tamoxifen regardless of their metabolizer status. It could be that CYP2D6 genotype–guided tamoxifen dose adjustment, in combination with concentration monitoring of tamoxifen metabolites, will maximize tamoxifen efficacy while maintaining patients’ quality of life and adherence to therapy.

## Figures and Tables

**TABLE A1.**
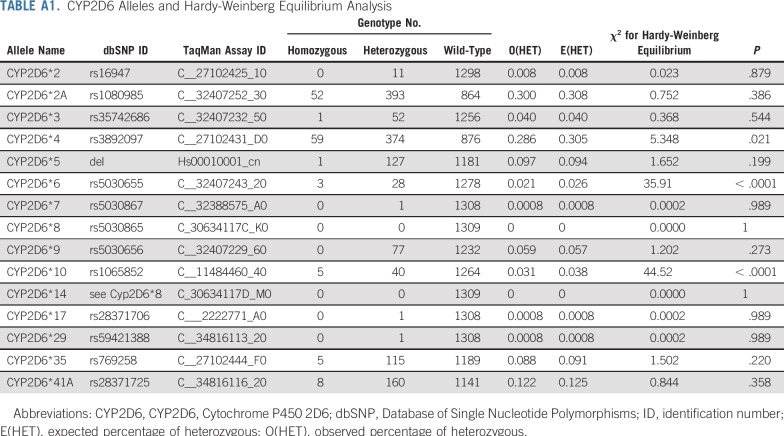
CYP2D6 Alleles and Hardy-Weinberg Equilibrium Analysis

**TABLE A2.**
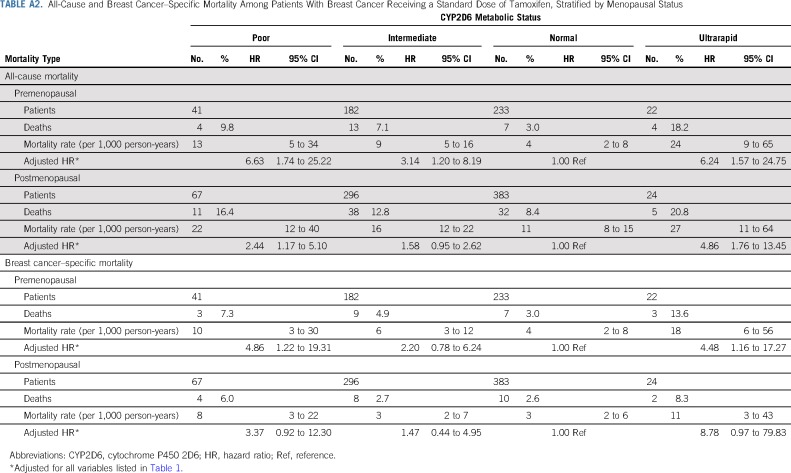
All-Cause and Breast Cancer–Specific Mortality Among Patients With Breast Cancer Receiving a Standard Dose of Tamoxifen, Stratified by Menopausal Status

## References

[1] Early Breast Cancer Trialists’ Collaborative Group (EBCTCG) Effects of chemotherapy and hormonal therapy for early breast cancer on recurrence and 15-year survival: An overview of the randomised trials Lancet 365 1687 1717 2005 15894097 10.1016/S0140-6736(05)66544-0

[2] Osborne CK Tamoxifen in the treatment of breast cancer N Engl J Med 339 1609 1618 1998 9828250 10.1056/NEJM199811263392207

[3] Hoskins JM Carey LA McLeod HL CYP2D6 and tamoxifen: DNA matters in breast cancer Nat Rev Cancer 9 576 586 2009 19629072 10.1038/nrc2683

[4] Goetz MP Rae JM Suman VJ et al Pharmacogenetics of tamoxifen biotransformation is associated with clinical outcomes of efficacy and hot flashes J Clin Oncol 23 9312 9318 2005 16361630 10.1200/JCO.2005.03.3266

[5] Goetz MP Suman VJ Hoskin TL et al CYP2D6 metabolism and patient outcome in the Austrian Breast and Colorectal Cancer Study Group trial (ABCSG) 8 Clin Cancer Res 19 500 507 2013 23213055 10.1158/1078-0432.CCR-12-2153 PMC3548984

[6] Kiyotani K Mushiroda T Imamura CK et al Significant effect of polymorphisms in CYP2D6 and ABCC2 on clinical outcomes of adjuvant tamoxifen therapy for breast cancer patients J Clin Oncol 28 1287 1293 2010 20124171 10.1200/JCO.2009.25.7246 PMC4872305

[7] Schroth W Goetz MP Hamann U et al Association between CYP2D6 polymorphisms and outcomes among women with early stage breast cancer treated with tamoxifen JAMA 302 1429 1436 2009 19809024 10.1001/jama.2009.1420 PMC3909953

[8] Regan MM Leyland-Jones B Bouzyk M et al CYP2D6 genotype and tamoxifen response in postmenopausal women with endocrine-responsive breast cancer: The Breast International Group 1-98 trial J Natl Cancer Inst 104 441 451 2012 22395644 10.1093/jnci/djs125 PMC3309132

[9] Rae JM Drury S Hayes DF et al CYP2D6 and UGT2B7 genotype and risk of recurrence in tamoxifen-treated breast cancer patients J Natl Cancer Inst 104 452 460 2012 22395643 10.1093/jnci/djs126 PMC3611934

[10] Goetz MP Ratain M Ingle JN Providing balance in ASCO clinical practice guidelines: CYP2D6 genotyping and tamoxifen efficacy J Clin Oncol 34 3944 3945 2016 27551126 10.1200/JCO.2016.68.5214

[11] Goetz MP Sangkuhl K Guchelaar HJ et al Clinical Pharmacogenetics Implementation Consortium (CPIC) guideline for CYP2D6 and tamoxifen therapy Clin Pharmacol Ther 103 770 777 2018 29385237 10.1002/cpt.1007 PMC5931215

[12] Saladores P Mürdter T Eccles D et al Tamoxifen metabolism predicts drug concentrations and outcome in premenopausal patients with early breast cancer Pharmacogenomics J 15 84 94 2015 25091503 10.1038/tpj.2014.34 PMC4308646

[13] Schroth W Winter S Mürdter T et al Improved prediction of endoxifen metabolism by CYP2D6 genotype in breast cancer patients treated with tamoxifen Front Pharmacol 8 582 2017 28955222 10.3389/fphar.2017.00582 PMC5609540

[14] Rolla R Vidali M Meola S et al Side effects associated with ultrarapid cytochrome P450 2D6 genotype among women with early stage breast cancer treated with tamoxifen Clin Lab 58 1211 1218 2012 23289191

[15] Holm J Eriksson L Ploner A et al Assessment of breast cancer risk factors reveals subtype heterogeneity Cancer Res 77 3708 3717 2017 28512241 10.1158/0008-5472.CAN-16-2574

[16] Holm J Humphreys K Li J et al Risk factors and tumor characteristics of interval cancers by mammographic density J Clin Oncol 33 1030 1037 2015 25646195 10.1200/JCO.2014.58.9986

[17] Gabrielson M Eriksson M Hammarström M et al Cohort profile: The Karolinska Mammography Project for Risk Prediction of Breast Cancer (KARMA) Int J Epidemiol 46 1740 1741g 2017 28180256 10.1093/ije/dyw357 PMC5837703

[18] Eriksson L He W Eriksson M et al Adjuvant therapy and mammographic density changes in women with breast cancer JNCI Cancer Spectr 2 pky071 2019 31360886 10.1093/jncics/pky071 PMC6649795

[19] Brand JS Li J Humphreys K et al Identification of two novel mammographic density loci at 6Q25.1 Breast Cancer Res 17 75 2015 26036842 10.1186/s13058-015-0591-2 PMC4501298

[20] Sideras K Ingle JN Ames MM et al Coprescription of tamoxifen and medications that inhibit CYP2D6 J Clin Oncol 28 2768 2776 2010 20439629 10.1200/JCO.2009.23.8931 PMC2881853

[21] Bradford LD CYP2D6 allele frequency in European Caucasians, Asians, Africans and their descendants Pharmacogenomics 3 229 243 2002 11972444 10.1517/14622416.3.2.229

[22] Gaedigk A Simon SD Pearce RE et al The CYP2D6 activity score: Translating genotype information into a qualitative measure of phenotype Clin Pharmacol Ther 83 234 242 2008 17971818 10.1038/sj.clpt.6100406

[23] He W Fang F Varnum C et al Predictors of discontinuation of adjuvant hormone therapy in patients with breast cancer J Clin Oncol 33 2262 2269 2015 26033800 10.1200/JCO.2014.59.3673

[24] He W Smedby KE Fang F et al Treatment restarting after discontinuation of adjuvant hormone therapy in breast cancer patients J Natl Cancer Inst 109 2017 10.1093/jnci/djx041 28423398

[25] Loprinzi CL Sloan J Stearns V et al Newer antidepressants and gabapentin for hot flashes: An individual patient pooled analysis J Clin Oncol 27 2831 2837 2009 19332723 10.1200/JCO.2008.19.6253 PMC2698018

[26] Nelson HD Vesco KK Haney E et al Nonhormonal therapies for menopausal hot flashes: Systematic review and meta-analysis JAMA 295 2057 2071 2006 16670414 10.1001/jama.295.17.2057

[27] Loprinzi CL Zahasky KM Sloan JA et al Tamoxifen-induced hot flashes Clin Breast Cancer 1 52 56 2000 11899390 10.3816/cbc.2000.n.004

[28] He W Eriksson L Törnberg S et al Discontinuation of adjuvant hormone therapy among breast cancer patients not previously attending mammography screening BMC Med 17 24 2019 30700300 10.1186/s12916-019-1252-6 PMC6354407

[29] Lambert PC Royston P Further development of flexible parametric models for survival analysis Stata J 9 265 290 2009

[30] Mürdter TE Schroth W Bacchus-Gerybadze L et al Activity levels of tamoxifen metabolites at the estrogen receptor and the impact of genetic polymorphisms of phase I and II enzymes on their concentration levels in plasma Clin Pharmacol Ther 89 708 717 2011 21451508 10.1038/clpt.2011.27

[31] Sanchez-Spitman A Dezentjé V Swen J et al Tamoxifen pharmacogenetics and metabolism: Results from the prospective CYPTAM study J Clin Oncol 37 636 646 2019 30676859 10.1200/JCO.18.00307

[32] Binkhorst L Mathijssen RH Jager A et al Individualization of tamoxifen therapy: Much more than just CYP2D6 genotyping Cancer Treat Rev 41 289 299 2015 25618289 10.1016/j.ctrv.2015.01.002

[33] Zanger UM Raimundo S Eichelbaum M Cytochrome P450 2D6: Overview and update on pharmacology, genetics, biochemistry Naunyn Schmiedebergs Arch Pharmacol 369 23 37 2004 14618296 10.1007/s00210-003-0832-2

[34] Love RR Desta Z Flockhart D et al CYP2D6 genotypes, endoxifen levels, and disease recurrence in 224 Filipino and Vietnamese women receiving adjuvant tamoxifen for operable breast cancer Springerplus 2 52 2013 23476897 10.1186/2193-1801-2-52 PMC3584248

[35] Ingelman-Sundberg M Genetic polymorphisms of cytochrome P450 2D6 (CYP2D6): Clinical consequences, evolutionary aspects and functional diversity Pharmacogenomics J 5 6 13 2005 15492763 10.1038/sj.tpj.6500285

[36] Owusu C Buist DS Field TS et al Predictors of tamoxifen discontinuation among older women with estrogen receptor-positive breast cancer J Clin Oncol 26 549 555 2008 18071188 10.1200/JCO.2006.10.1022

[37] Hertz DL Rae JM One step at a time: CYP2D6 guided tamoxifen treatment awaits convincing evidence of clinical validity Pharmacogenomics 17 823 826 2016 27249031 10.2217/pgs-2016-0059

[38] Rae JM Sikora MJ Henry NL et al Cytochrome P450 2D6 activity predicts discontinuation of tamoxifen therapy in breast cancer patients Pharmacogenomics J 9 258 264 2009 19421167 10.1038/tpj.2009.14 PMC2991048

[39] Brauch H Schroth W Goetz MP et al Tamoxifen use in postmenopausal breast cancer: CYP2D6 matters J Clin Oncol 31 176 180 2013 23091108 10.1200/JCO.2012.44.6625 PMC3731938

[40] Torre LA Bray F Siegel RL et al Global cancer statistics, 2012 CA Cancer J Clin 65 87 108 2015 25651787 10.3322/caac.21262

[41] Gaedigk A Sangkuhl K Whirl-Carrillo M et al Prediction of CYP2D6 phenotype from genotype across world populations Genet Med 19 69 76 2017 27388693 10.1038/gim.2016.80 PMC5292679

[42] Sinha G Gene testing to tailor breast cancer therapy has arrived: Is it ready for the clinic? J Natl Cancer Inst 100 1050 1051 2008 18664643 10.1093/jnci/djn286

